# Thyroid surgery in children: a single pediatric center experience

**DOI:** 10.1007/s13304-026-02864-8

**Published:** 2026-09-25

**Authors:** Giovanni Conzo, Carmine Noviello, Mercedes Romano, Ilaria Cascone, Gaia De Francesco, Giovanni Docimo, Anna Grandone, Alfonso Papparella

**Affiliations:** 1 Division of General and Oncological Surgery, Department of Translational Medical Sciences, University of Campania ‘Luigi Vanvitelli’ Naples Italy, Naples, Italy; 2 Pediatric Surgery Unit, Department of Woman, Child, General and Specialized Surgery, University of Campania “Luigi Vanvitelli”, Naples, Italy; 3 Department of Woman, Child, General and Specialized Surgery, University of Campania “Luigi Vanvitelli” Largo Madonna delle Grazie, Napoli, 1 80138 Italy

**Keywords:** Children, Pediatric thyroidectomy, Thyroid carcinoma, Multidisciplinary team, Pediatric surgeon, Thyroid disease

## Abstract

The increasing incidence of thyroid disease in children requires appropriate training for pediatric surgeons. The specific characteristics of this age group require a different treatment approach from that used in adults.

The authors report their pediatric experience over a 5-year period. For each patient, we analyzed the indication for surgery, type of procedure, postoperative complications, and follow-up, with a mean follow-up of one year. The mean age of the 25 patients treated was 12 years (range, 5–16 years), and the male-to-female ratio was 1:1.27. The most common clinical presentation was asymptomatic neck swelling or an incidental finding of a thyroid nodule, often detected during evaluation for weight loss or follow-up for another condition. All patients with thyroid nodules underwent preoperative fine-needle aspiration, and surgery was indicated in cases of malignancy. Four patients were diagnosed with Graves’ disease. Surgical treatment was performed by a pediatric surgeon and a general surgeon. All patients underwent total thyroidectomy, and three underwent lymph node dissection. The mean operative time was 80.7 min (range, 61.0–100.4 min). Histopathological examination of the surgical specimens showed 22 cases of carcinoma (18 papillary and 4 follicular) and 3 cases of autoimmune thyroid disease. The mean hospital stay was 4 days (range, 3–6 days). Transient asymptomatic hypocalcemia was observed in seven patients (28%). Preoperative laryngoscopy identified unilateral vocal cord paralysis in two patients (8%); these abnormalities were present before surgery and were therefore not considered surgical complications. Postoperative radioiodine treatment was administered to three patients. Pediatric endocrinologists conducted postoperative follow-up, including clinical assessment, ultrasound, and laboratory tests. Thyroidectomy in children should be performed in a specialized multidisciplinary pediatric center, with the involvement of an experienced thyroid surgeon when appropriate.

**Graphical abstract:**

**Figure:**
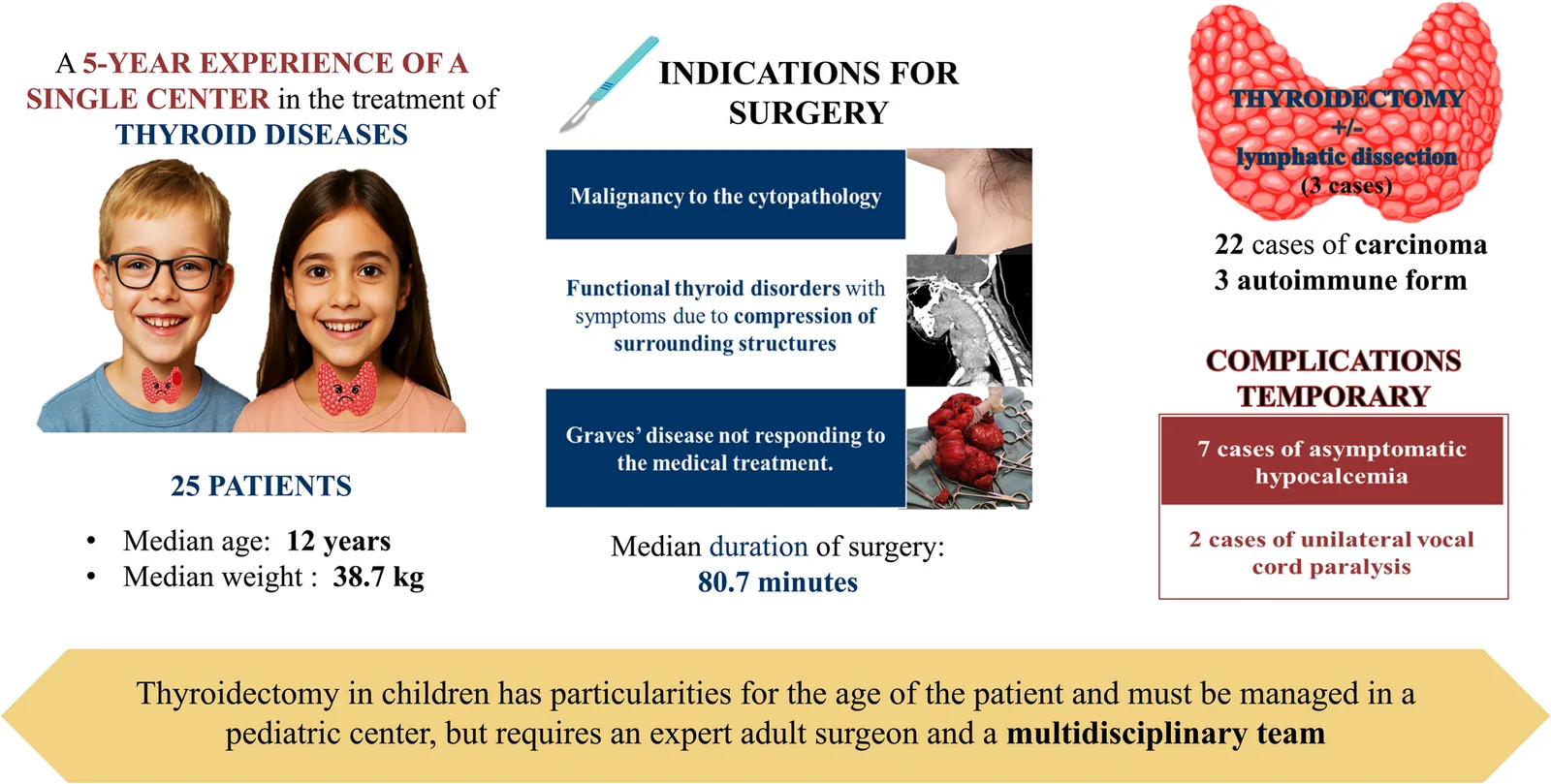

## Introduction

In recent decades, although thyroid disease requiring surgery remains uncommon in children, the incidence of pediatric differentiated thyroid carcinoma has increased [1–3]. Consequently, pediatric surgeons play an important role in the diagnosis and treatment of these conditions. The estimated prevalence of solid thyroid nodules in the pediatric population is 1–1.7% [4], with a higher prevalence among females. The risk of malignancy in a solitary pediatric thyroid nodule ranges from 19% to 26.4% [5–7], which is higher than the approximately 5% reported in adults. Furthermore, experience is particularly important in this age group [8].

The lack of specific guidelines for pediatric thyroid nodules before 2015 resulted in the use of adult standards in children. However, pediatric patients more often present with advanced disease at diagnosis, including increased lymph node involvement, distant metastases, and multifocal disease. Specific guidelines for the diagnosis, treatment, and follow-up of pediatric thyroid nodules and differentiated thyroid cancer have therefore been developed by the American Thyroid Association (ATA) and the European Thyroid Association (ETA) [4, 9]. Nevertheless, the prognosis of pediatric differentiated thyroid cancer is excellent despite its more aggressive presentation [10–12].

In pediatric patients, the most common clinical presentation is asymptomatic thyroid swelling. Less frequently, patients may present with cervical lymphadenopathy or a thyroid nodule detected on imaging or during evaluation for an unrelated condition [4]. As in adults, a detailed family history and physical examination are the first steps in the diagnostic process. Assessment of thyroid hormone levels and antithyroid antibodies, together with diagnostic imaging, completes the initial evaluation. Imaging studies include thyroid ultrasonography and, when indicated, scintigraphy, followed by fine-needle aspiration (FNA), usually performed under ultrasound guidance [9]. Surgical indications for thyroidectomy in pediatric patients include malignant cytology, autoimmune diseases such as Graves’ disease (GD), prophylactic treatment of medullary thyroid carcinoma (MTC) in patients with multiple endocrine neoplasia type 2 (MEN 2), and functional thyroid disorders causing compressive symptoms involving the trachea or esophagus [13].

The Tumor, Node, Metastasis (TNM) classification is used for staging. Papillary and follicular thyroid carcinomas are classified into two stages: stage I (any T, any N, M0) and stage II (any T, any N, M1) [4]. The diagnosis guides the choice of surgical approach; total thyroidectomy is frequently performed and may be accompanied by cervical lymph node dissection when indicated.

The most common complications after thyroid surgery include hypoparathyroidism, which may cause transient or permanent hypocalcemia, as well as bleeding, wound infection, and injury to the recurrent laryngeal nerves and vocal cords [14–16].

Because of the rarity of thyroid cancer, only a small number of children undergo thyroid surgery each year, and referral to an endocrine surgery center may require a substantial number of cases to develop extensive experience [17]. Long-term survival exceeds 98% at 30 years. The indications for total thyroidectomy (TT) versus lobectomy, prophylactic neck dissection, and the use of radioiodine remain subjects of ongoing debate.

For this reason, the question of which specialist should treat pediatric thyroid disease and whether these procedures should be performed exclusively in high-volume centers remains debated. We reviewed our experience over a 5-year period at a single low-volume pediatric center. To combine the benefits of high-volume thyroid surgery with specialized pediatric care, patients were treated by a multidisciplinary team including pediatric endocrinologists, pediatric surgeons, and an experienced high-volume thyroid surgeon.

Therefore, the primary aim of this retrospective study was to evaluate the feasibility and short-term safety of thyroid surgery in children performed at a low-volume pediatric center within a multidisciplinary model involving pediatric surgeons, pediatric endocrinologists, and an experienced high-volume thyroid surgeon. Secondary objectives were to describe the clinical characteristics, surgical indications and procedures, postoperative complications, and length of hospital stay in our cohort. Our results were compared with those reported in the literature.

## Methods

A retrospective study was conducted in patients younger than 16 years who underwent thyroid surgery at a pediatric surgery center. All patient management, from diagnosis to surgical treatment and follow-up, was performed by a multidisciplinary team consisting of a pediatric surgeon, a pediatric endocrinologist, and a surgeon with expertise in adult thyroid surgery. The study period was 5 years, from January 2020 to December 2024. After suspicion of thyroid disease, all patients underwent endocrinological evaluation, blood tests for thyroid hormones and antithyroid antibodies, neck ultrasonography with nodule classification according to EU-TIRADS, and FNA for solid or partially solid nodules larger than 1.0 cm or nodules larger than 0.5 cm in patients with a family history of thyroid cancer. FNA under ultrasound guidance was performed by cytopathologists. In younger children (under 6 years of age), general anesthesia was used; in older children, local anesthesia was used.

During the study period, 95 FNAs were performed at the two participating centers in children evaluated for thyroid nodules. Patients with malignant cytology were referred to our pediatric center for surgical treatment. Because the present study was based retrospectively on the surgical cohort, complete data regarding the clinical course and management of all children undergoing FNA at the two centers were not available. Therefore, the present analysis includes the 25 patients who underwent thyroid surgery at our center during the study period and met the predefined inclusion criteria.

Demographic data (age, sex, and weight) and clinical data (comorbidities, preoperative diagnosis, preoperative serum PTH, calcium, phosphorus, and magnesium levels, neck ultrasonography, CT findings, and TNM stage) were collected. In cases of dysphonia, preoperative laryngoscopy was performed. Calcium and vitamin D supplementation was administered before surgery when necessary to achieve normal serum levels. Surgical data, including operative time and short-term complications, as well as length of hospital stay, were collected. Indications for surgery included malignant cytology according to the Bethesda system, functional thyroid disorders causing compressive symptoms, or autoimmune diseases such as GD that did not respond to medical treatment after at least 18 months.

After surgery, blood tests were performed at 8, 12, and 24 h to assess serum calcium, PTH, and thyroid hormone levels. Asymptomatic transient hypocalcemia was defined as a postoperative serum calcium level, corrected for serum albumin, of < 8.0 mg/dL in at least one measurement.

Intravenous tramadol, ondansetron, and ketorolac were administered during the first 24 h to provide analgesia and prevent postoperative nausea and vomiting. All patients had a mean follow-up of one year, provided by pediatric endocrinologists and, for patients diagnosed with carcinoma, pediatric oncologists.

Radioactive iodine (RAI) was recommended as adjuvant therapy for patients with lymph node involvement.

## Results

A total of 25 patients (11 males) with benign or malignant thyroid conditions requiring thyroidectomy were included. The mean age at surgery was 12 years (range, 5–16 years), and the male-to-female ratio was 1:1.27. The mean weight at surgery was 38.7 kg (range, 25.0–57.3 kg), and 15 patients (60% of the cohort) weighed < 30 kg. In most cases (20 patients), the clinical presentation was asymptomatic swelling due to a thyroid nodule involving one side of the neck (13 on the right). Three patients had swelling of the entire thyroid gland (goiter), associated with dysphonia. In two cases, a thyroid nodule was incidentally discovered during investigations for other reasons, such as obesity (Table 1). Three patients had type 1 diabetes mellitus, and one patient had DICER1 syndrome and a previous renal operation for Wilms tumor.

**Table 1:** Clinical presentation of the patients with fine-needle aspiration (FNA), malignancy and Graves’ Disease (GD)

Clinical presentation	Cases	FNA	Malignancy GD	
asymptomatic swelling of right neck	13	13	13	0
asymptomatic swelling of left neck	7	7	7	0
swelling of the entire thyroid gland	3	3	0	3
Dysphonia	2	2		
No dysphonia	1	1		
Thyroid nodule found during other tests	2	2	2	1

All patients underwent blood tests, including FT3, FT4, TSH, anti-TSHR, anti-TPO, and anti-TG antibodies, as well as vitamin D, calcium, phosphorus, and magnesium levels. All patients underwent neck ultrasonography and FNA (21 unilateral and 4 bilateral procedures). The preoperative diagnostic work-up included neck ultrasonography with nodule classification according to the EU-TIRADS system, neck CT in cases of FNA-diagnosed malignancy, and laryngoscopy when dysphonia was clinically evident. During the study period, a total of 95 FNA procedures for thyroid nodules were performed at the two participating centers.

The most frequent indication for surgery was malignancy, which was present in 22 cases (88%), including one patient with GD and a thyroid nodule. Two patients underwent surgery for GD associated with laryngeal compression (dysphonia), while the remaining patient had GD that failed to remit despite 20 months of medical treatment. The three patients with GD received medical treatment before surgery because of persistently elevated thyroid hormone levels. All patients with thyroid carcinoma were classified as TNM stage I. Cervical lymph node involvement was present in three cases; these patients received adjuvant RAI treatment. No distant metastases were observed.

**Fig. 1:**
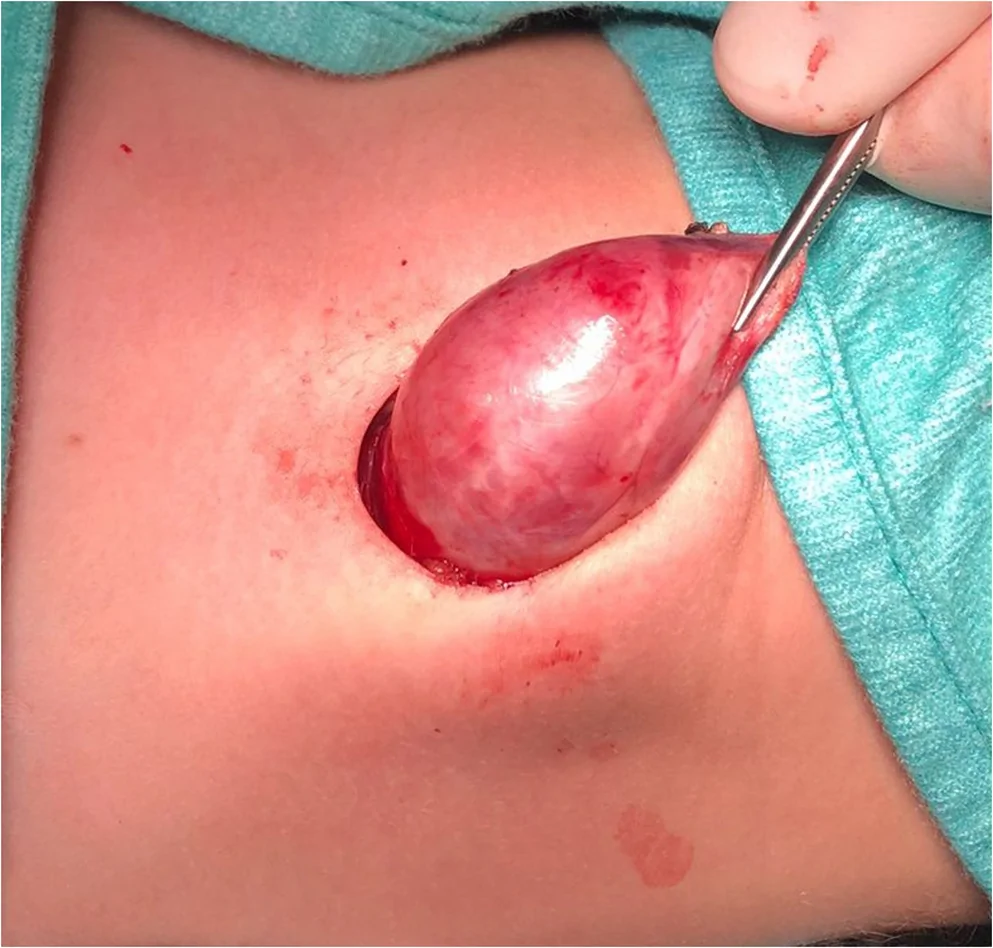
Surgical image of excised thyroid cancer

**Fig. 2:**
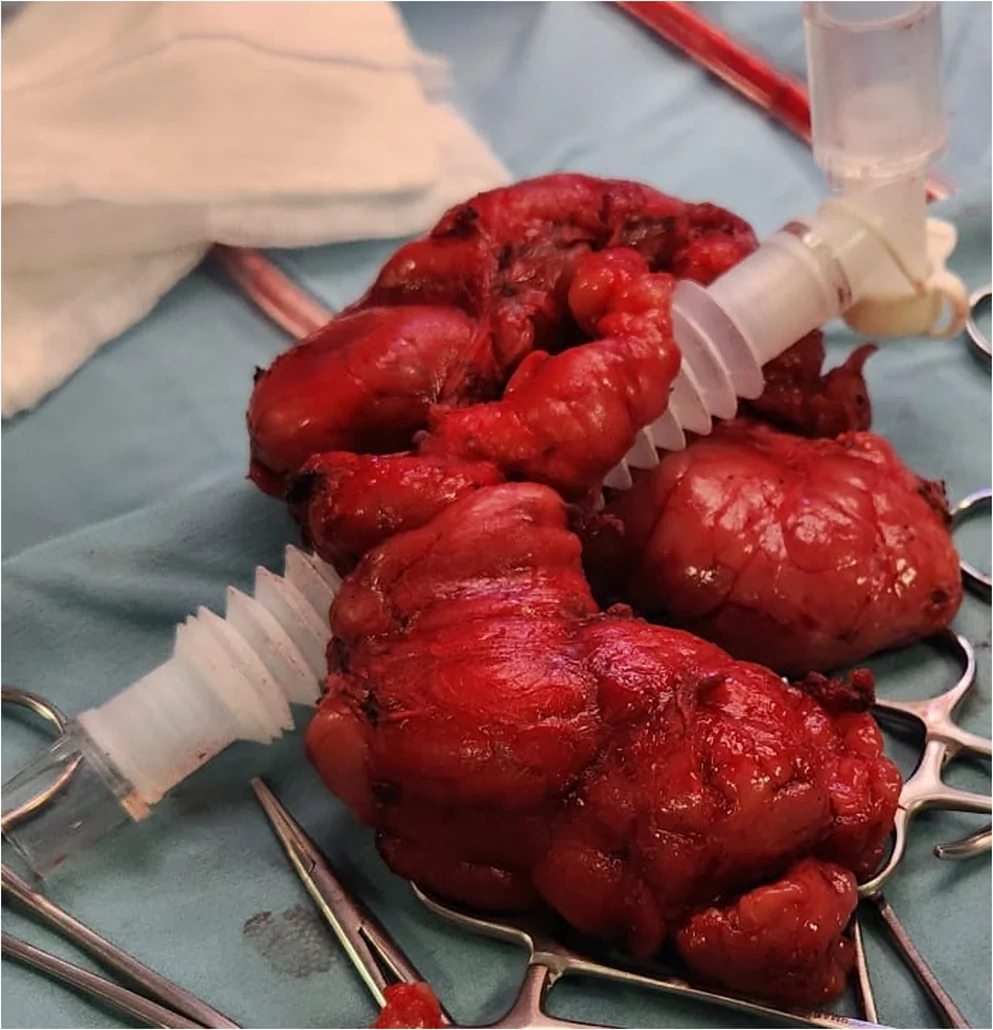
Surgical image of thyroid goiter removed. The white tube shows the relationship of the large thyroid with the trachea

All patients underwent total thyroidectomy (Fig. 1). Three patients underwent lymph node dissection, one of whom required a second lymph node dissection 3 months later. In one case, a large goiter was removed (Fig. 2). In all surgical procedures, the parathyroid glands were identified and preserved. The mean operative time was 80.7 min (range, 61.0–100.4 min). Patients with GD and synchronous lymph node involvement of the central and lateral cervical compartments required a longer operative time.

Among the 25 patients, 22 (88%) had malignant thyroid disease, including 18 patients with papillary thyroid carcinoma (72% of the total cohort) and 4 with follicular thyroid carcinoma (16% of the total cohort). The remaining three patients (12%) had autoimmune thyroid disease without malignancy. Among the 22 malignant cases, papillary thyroid carcinoma accounted for 81.8% and follicular thyroid carcinoma for 18.2%.

Hemostatic materials were required in some patients because of bleeding from the surgical bed. A surgical drain was placed in 17 patients (68%), left in place for 48 h, and subsequently removed. Oral feeding was initiated on postoperative day 2 in all cases. The mean hospital stay was 4 days (range, 3–6 days). In two cases, preoperative vocal cord paralysis was identified by laryngoscopy. The only postoperative complication observed was asymptomatic transient hypocalcemia, which occurred in 7 patients (28%) and resolved within 4 days. No rehospitalizations, surgical wound infections, or postoperative hematomas were reported. All patients were followed by pediatric endocrinologists and, when appropriate, by pediatric oncologists.

## Discussion

The risk of thyroid cancer in children and adolescents is remarkably low compared with that in older populations [18]. However, thyroid cancer is the most common pediatric endocrine neoplasm, accounting for approximately 3% of all pediatric malignant neoplasms and 5–5.7% of malignant neoplasms of the head and neck. Most pediatric thyroid cancers are differentiated, with papillary thyroid carcinoma (PTC) accounting for the vast majority, followed by follicular and medullary carcinomas [19]. Pediatric thyroid cancers are more frequently metastatic at diagnosis than adult thyroid cancers but are associated with an excellent prognosis and very low disease-specific mortality [17–19].

Given the low incidence of thyroid cancer in children, it remains unclear whether management should be performed by adult or pediatric specialists [20–22]. Before the publication of the 2015 ATA guidelines, the management of pediatric thyroid nodules and thyroid cancer primarily followed adult guidelines [4]. Children with thyroid cancer were traditionally treated with total thyroidectomy followed by radioactive iodine (RAI) ablation. Although this approach achieved high cure rates, long-term follow-up raised concerns about the potential late adverse effects of radiation exposure [23]. Children have anatomical features that distinguish them from adults and may have a higher risk of post-thyroidectomy complications, particularly hypocalcemia and recurrent laryngeal nerve injury [14–16]. Respiratory and infectious complications, as well as bleeding, are other potential complications of thyroid surgery in children [23–25].In recent years, the availability of pediatric-specific molecular and clinical data has increased significantly, allowing a more targeted approach to the management of pediatric patients [17]. One of the main goals of the 2015 ATA pediatric guidelines was to establish a more precise management strategy and limit the use of radioiodine. Other key differences from adult guidelines included a lower threshold for FNA of thyroid nodules and the recommendation of lobectomy rather than repeat FNA for indeterminate cytology [4, 24, 26]. Long-term follow-up studies have reported a lower incidence of local recurrence after total thyroidectomy than after lobectomy (6% vs. 35%) [23–31]. Total thyroidectomy also facilitates the use of radioactive iodine for the detection and treatment of residual disease and enables serum thyroglobulin measurement for long-term surveillance.

Because of the multifocality of pediatric thyroid carcinoma [4, 28–32], pediatric guidelines recommend total or near-total thyroidectomy in selected cases. Current guidelines recommend therapeutic central neck dissection in the presence of clinically evident central compartment metastases, whereas lateral neck dissection is generally reserved for confirmed lateral lymph node metastases. Children are more likely to have lymph node involvement and lung metastases at diagnosis [11, 24, 25]. This, however, is offset by an excellent overall prognosis [17, 18, 24].

According to available epidemiological data, new cases in individuals younger than 20 years account for approximately 1.8% of all thyroid cancers [12]. Thyroid cancer in children differs substantially from adult disease in terms of clinical presentation, management, and treatment costs. The ATA guidelines define an experienced surgeon as one who performs more than 30 procedures annually. However, this benchmark is difficult for pediatric surgeons to meet because of the rarity of pediatric thyroid surgery. Pediatric surgeons often perform only a small number of thyroid procedures per year, making it unlikely that they will meet the high-volume criteria established for this specialty [24]. Conversely, although an adult surgeon with expertise in thyroid surgery has extensive experience with thyroid procedures, they may lack the specific expertise required to manage pediatric thyroid disease. Therefore, at our center, thyroid disease is managed by a multidisciplinary team consisting of a pediatric surgeon and a general surgeon with expertise in thyroid surgery.

We evaluated the feasibility and safety of thyroid surgery in our low-volume pediatric center and compared our results with those reported in the literature [26, 27]. No major differences were observed in terms of operative time or hospital stay. In our cohort, transient asymptomatic hypocalcemia occurred in seven patients (28%), which is consistent with the incidence reported in the literature (15–50%) [14–16]. None of our patients developed permanent hypocalcemia. Hypocalcemia appeared to be more frequent among children weighing less than 30 kg and among those undergoing more extensive surgery or lymph node dissection; however, these observations should be interpreted cautiously given the small sample size and the limited number of events. Similarly, lower postoperative PTH levels were observed in patients who developed hypocalcemia, but this finding should be considered exploratory and cannot establish an independent association in this small cohort.

In our series, hypocalcemia was successfully managed with oral calcium supplementation and resolved within four days. No patient required intravenous calcium gluconate, and no cases of permanent hypoparathyroidism were observed. Perioperative PTH assessment may be useful for postoperative monitoring and may contribute to the management of patients at risk of hypocalcemia. As a preventive measure, patients with hypocalcemia or those considered at high risk received calcium and vitamin D supplementation before surgery. In our cohort, transient asymptomatic hypocalcemia was associated with a longer hospital stay; however, this finding should be interpreted cautiously given the small sample size.

Preoperative euthyroidism was observed in 22 patients, while three patients with GD required medical treatment before surgery. No patient experienced permanent complications. No surgical wound infections occurred. No local or distant recurrence was observed during the available follow-up period.

This study has several limitations, including its retrospective design, small sample size, single-center surgical cohort, lack of a complete denominator for all children evaluated for thyroid nodules, absence of a comparison group, and relatively short follow-up (mean approximately one year). These factors may have introduced selection bias, limit the generalizability of our findings, and preclude definitive assessment of the superiority of our multidisciplinary model or of long-term oncological and endocrine outcomes. The study should therefore be interpreted as a description of our center’s surgical experience rather than as an assessment of the management of the entire pediatric thyroid population.

## Conclusion

Children are not “little adults”: they have an excellent prognosis, and overtreatment should be avoided whenever possible. Risk stratification allows identification of appropriate indications for total thyroidectomy, neck dissection, and adjuvant RAI treatment in selected cases. Given the multifocal nature of pediatric thyroid carcinoma, lobectomy should be reserved for low-risk cases. At the same time, minimizing local recurrence is important to reduce the risk of complications associated with reoperation.

In our experience, the involvement of an experienced high-volume thyroid surgeon represents a feasible approach to the management of pediatric thyroid disease in a low-volume pediatric center. This case series should be interpreted as an observational description of our institutional experience; we cannot establish the superiority or necessity of this multidisciplinary model compared with alternative organizational approaches.
